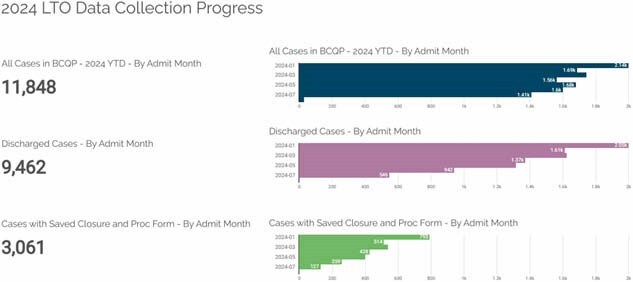# 974 Implementation of Long-term Outcomes for Burn Patients in BCQP – Lessons Learned

**DOI:** 10.1093/jbcr/iraf019.505

**Published:** 2025-04-01

**Authors:** Claudia Malic, Joan Weber, Adam Singer, Bethany Schmoker, Melissa O’Connor, Heidi Altamirano, Thereasa Abrams, Samuel Mandell, Nicole Bernal, David Harrington

**Affiliations:** Ottawa Children’s Hospital of Eastern Ottawa; American Burn Association; Stony Brook University; University of Colorado Hospital; MedStar Washington Hospital Center; Regions Hospital; University of Tennessee, Knoxville; University of Texas Southwestern / Parkland; Ohio State University, Wexner Medical Center; Rhode Island Burn Center

## Abstract

**Introduction:**

Burn registries systematically collect burn-specific data to better understand the epidemiology, clinical care and outcomes. However, there is a gap in data regarding outcomes as patients transition from inpatient to outpatient care. Collecting burn injury-related data post-discharge can inform clinicians and improve long-term outcomes.

In January 2024, the American Burn Association’s Burn Care Quality Platform (BCQP) implemented variables for long-term outcomes (LTO) for patients transitioning to outpatient follow-up after inpatient discharge.

**Methods:**

The data for the period January 1 – July 31st, 2024, was retrieved from BCQP. The software provider analyzed the data and provided a report of the burn centers who submitted data for LTOs for burn patients who were admitted. The variables were available only for centers who uses the full platform. Data interpretation and visualization were reviewed, and feedback was provided to the software provider to improve the readability of such reports.

**Results:**

Over seven months, 67 burn centers entered 9,124 discharged cases; 3,061 had wound closure variables documented. Forty-three centers completed follow-up on over 10% of their discharges, with 32 centers documenting more than 50%. Follow-up was documented in 2,360 cases, with completion in 634 (39 centers) and 1,248 ongoing. A total of 176 surgical procedures were performed in 122 unique cases, mostly as outpatients. Clinical evaluation was the most frequent method of scar assessment (1,870 records), followed by the Vancouver scale (402) and modified scale (211). Documentation of depression, PTSD, and ASD was minimal, while itch was noted in 1,516 records. Changes for documenting pain were introduced; 5,513 records for 1,880 unique cases from 42 centers showed 1,570 records with pain requiring prescriptions, 890 needing over-the-counter medication, and 219 still in pain but without medication. In 1670 records the pain was not present. Only in 899 records the presence of pain was not documented.

**Conclusions:**

There was a high uptake of documented LTOs after patients were discharged from the burn centers. Initial data reveals the importance of collecting data on outpatients to better understand long term outcomes of patients with burn injuries. All variables will be reviewed at the end of the year based on the feedback received from registrars on open house sessions.

**Applicability of Research to Practice:**

collection of the long terms outcomes for patients after discharge from their burn centers is essential and will provide further information about the impact that burn injuries has on long term on patients and their families

**Funding for the Study:**

N/A